# De Novo Foliar Transcriptome of *Chenopodium amaranticolor* and Analysis of Its Gene Expression During Virus-Induced Hypersensitive Response

**DOI:** 10.1371/journal.pone.0045953

**Published:** 2012-09-28

**Authors:** Yongqiang Zhang, Xinwu Pei, Chao Zhang, Zifeng Lu, Zhixing Wang, Shirong Jia, Weimin Li

**Affiliations:** 1 Biotechnology Research Institute, Chinese Academy of Agricultural Sciences, Beijing, People's Republic of China; 2 State Key Laboratory of Grassland Agro-ecosystems, College of Pastoral Agriculture Science and Technology, Lanzhou University, Lanzhou, People's Republic of China; Auburn University, United States of America

## Abstract

**Background:**

The hypersensitive response (HR) system of *Chenopodium* spp. confers broad-spectrum virus resistance. However, little knowledge exists at the genomic level for *Chenopodium*, thus impeding the advanced molecular research of this attractive feature. Hence, we took advantage of RNA-seq to survey the foliar transcriptome of *C. amaranticolor*, a *Chenopodium* species widely used as laboratory indicator for pathogenic viruses, in order to facilitate the characterization of the HR-type of virus resistance.

**Methodology and Principal Findings:**

Using Illumina HiSeq™ 2000 platform, we obtained 39,868,984 reads with 3,588,208,560 bp, which were assembled into 112,452 unigenes (3,847 clusters and 108,605 singletons). BlastX search against the NCBI NR database identified 61,698 sequences with a cut-off E-value above 10^−5^. Assembled sequences were annotated with gene descriptions, GO, COG and KEGG terms, respectively. A total number of 738 resistance gene analogs (RGAs) and homology sequences of 6 key signaling proteins within the R proteins-directed signaling pathway were identified. Based on this transcriptome data, we investigated the gene expression profiles over the stage of HR induced by *Tobacco mosaic virus* and *Cucumber mosaic virus* by using digital gene expression analysis. Numerous candidate genes specifically or commonly regulated by these two distinct viruses at early and late stages of the HR were identified, and the dynamic changes of the differently expressed genes enriched in the pathway of plant-pathogen interaction were particularly emphasized.

**Conclusions:**

To our knowledge, this study is the first description of the genetic makeup of *C. amaranticolor*, providing deep insight into the comprehensive gene expression information at transcriptional level in this species. The 738 RGAs as well as the differentially regulated genes, particularly the common genes regulated by both TMV and CMV, are suitable candidates which merit further functional characterization to dissect the molecular mechanisms and regulatory pathways of the HR-type of virus resistance in *Chenopodium*.

## Introduction

During the entire lifetime, plants are attacked by a broad range of pathogens, being the targets of bacteria, fungi, viruses, protozoa as well as nematodes. To combat the ever-changing landscape of biotic interactions, plants have evolved sophisticated and effective defense mechanisms. The hypersensitive response (HR) represents one of the most active resistance responses. This type resistance often occurs following the incompatible interactions between plants and pathogens, and is characterized by rapid necrosis of infected and neighboring host cells [1], [2]. Regarding to the well known ‘Gene-for-Gene’ hypothesis [3], the HR depends on a genetic interaction, either directly or indirectly, between the product of a dominant or semidominant resistance (*R*) gene and a corresponding pathogen avirulence (*Avr*) gene product,thereby preventing pathogen growth and spread in plants [1], [4], [5].

The *R* gene-mediated HR usually is associated with rapid initiation of signal transduction pathways leading to expression of disease resistance responses, such as the oxidative burst, alteration of membrane potentials, increases in lipoxygenase activity, production of antimicrobial compounds, and the expression of defense-related genes [6]. Plants undergoing HR also induce a state of pathogen-nonspecific resistance throughout the plant, a phenomenon termed systemic acquired resistance [7]. This local response is therefore attractable in plant breeding programs, and characterization of host genetic elements involved in HR is assumed to have great potential for developing new strategies to reduce losses from plant disease. Over the past decades, the detailed analysis of many hypersensitive disease resistance systems has substantially advanced the basic research on HR-type of resistance mechanisms. Progress has been made in characterizing *R* genes as well as the signal-transduction events that coordinate the R protein-directed HR-type plant defense [4], [5], [8].

The HR system in *Chenopodium* spp. is one of the most notable disease resistance mechanisms. Many plant viruses in over a dozen of genera, including bromo-, como-, cucumo-, clostero-, potex-, poty- and tobamovirus have been documented to elicit local lesion HR on *Chenopodium* [9], [10]. The broad-spectrum virus resistance suggests *Chenopodium* are rich in genes that act in restricting viral spread as well as that confer multi-virus resistance to a plant. However, understanding of the HR-type of resistance in *Chenopodium* is surprisingly incomplete. Due to lack of developed genetic tools such as tissue culture and transformation, only few studies were performed to define the genetic mechanisms of HR in *Chenopodium* [10]–[13]. Genomic information on *Chenopodium* spp. is rather limited, only 1,249 expressed sequence tags (EST) and 358 proteins have been deposited in Genbank as of May 2012.

The next-generation sequencing, referred to as RNA sequencing (RNA-Seq), provides a unique opportunity for genomic exploration in non-model plant species that do not have a reference genome sequence data [14], [15]. So far, RNA-Seq has been successfully used for annotation, transcript profiling and single nucleotide polymorphism (SNP) discovery in a number of non-model plant species, such as alfalfa [16], *Artemisia annua* [17], buckwheat [18], California poppy [19], *Eucalyptus grandis* [20], grape [21], magnoliid avocado [19], orchid [22] and *Pachycladon enysii* [23].

**Figure 1:**
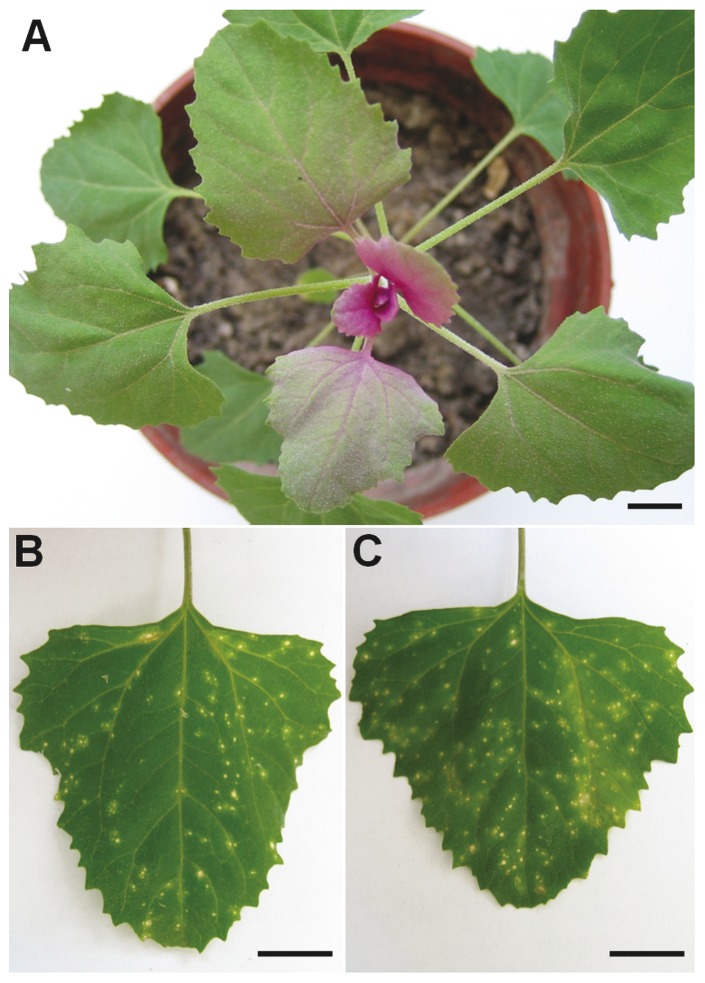
Virus induced local lesions on leaves of *C. amaranticolor*. (A) The 6-week old plant of *C. amaranticolor*. (B) Leaves inoculated with *Tobacco mosaic virus* at 40 hours p.i.. (C) Leaves inoculated with *Cucumber mosaic virus* at 40 hours p.i.. Scale bar = 1.0 cm.

In order to facilitate the investigation of the HR-type of virus resistance in *Chenopodium*, herein we took advantage of RNA-seq to survey the foliar transcriptome of *C. amaranticolor*, one *Chenopodium* species being most widely used as laboratory indicator for pathogenic viruses (Figure 1A). Over 3.5 gigabase pairs of high-quality DNA sequence were generated with Illumina technology. After *de novo* assembly and annotation, a sufficiently large transcriptome database containing 112,452 distinct sequences was built. By using Illumina's digital gene expression (DGE) platform, this database was immediately applied to analyze the gene expression profiles over the stage of HR induced by *Tobacco mosaic virus* (TMV) and *Cucumber mosaic virus* (CMV) (Figure 1B and 1C), two agronomically important but taxonomically distinct viruses [24]. The foliar transcriptome of *C. amaranticolor* combined with the DGE profiles lays the foundation for future functional genomics studies on *Chenopodium*, in particular for identification of genes involved in the complex signaling and regulation pathways that mediate the HR-type of virus resistance.

## Results and Discussion

### Illumina sequencing and reads assembly

To obtain an overview of the *C. amaranticolor* gene expression profile at leaves, a cDNA sample generated from the mixed materials, which contain equal amount of healthy leaves, TMV-inoculated leaves at 6 hours post inoculation (hpi) (TMV-6h) and 28 hpi (TMV-28h), and CMV-inoculated leaves at 6 hpi (CMV-6h) and 20 hpi (CMV-20h), were subjected to sequence by using the Illumina sequencing platform HiSeq™ 2000. Notably, under the greenhouse conditions in this study, the leaves inoculated with TMV at 28 hpi or with CMV at 20 hpi appeared just visible local lesions. After filtering for adaptors and low-quality sequences, 39,868,984 clean reads, each of which has the length of 90 bp, were generated (Table 1).

**Table 1:** General features of the foliar transcriptome of *C. amaranticolor*.

Total number of reads^a^	39,868,984
Total base pairs (bp)	3,588,208,560
Average read length (bp)	90
Total number of contigs	326,027
Mean length of contigs (bp)	163
Total number of scaffolds	172, 748
Mean length of scaffolds (bp)	253
Distinct clusters	3,848
Distinct singletons	108, 605
Total distinct sequences	112, 452
Sequences with E-value<10^−5^	62,482

a Adaptors and low-quality reads were excluded.

To facilitate sequence assembly, the raw reads were first randomly clipped into 21-mers using SOAPdenovo software [25]. These short 21-mers were then assembled into 326,027 contigs (Table 1) with average lengths of 163 bp, and their size distribution is shown in Figure S1A. Using paired-end joining and gap-filling, the contigs were assembled into 172,748 scaffolds with a mean size of 253 bp including 6,034 scaffolds larger than 800 bp (Table 1). These scaffolds were further analyzed by using TGICL software [26], resulting in 112,452 unigenes with 3,847 clusters (mean size: 575 bp) and 108,605 singletons (mean size: 307 bp) (Table 1). In this study, the singleton means a scaffold that failed to match other scaffolds and the unigenes include all the clusters and singletons generated from scaffolds. The size distribution of these distinct sequences is shown in Figure S1B. All files of assembled contigs and unigenes are available by request.

To verify the quality of sequencing data, 12 unigenes were randomly selected and pairs of primers were designed accordingly for RT-PCR amplification. Consequently, each of primer pairs generated a band with the expected size and the identity of all 12 PCR products were confirmed by Sanger sequencing (data not shown).

### Annotation of predicted proteins

**Figure 2:**
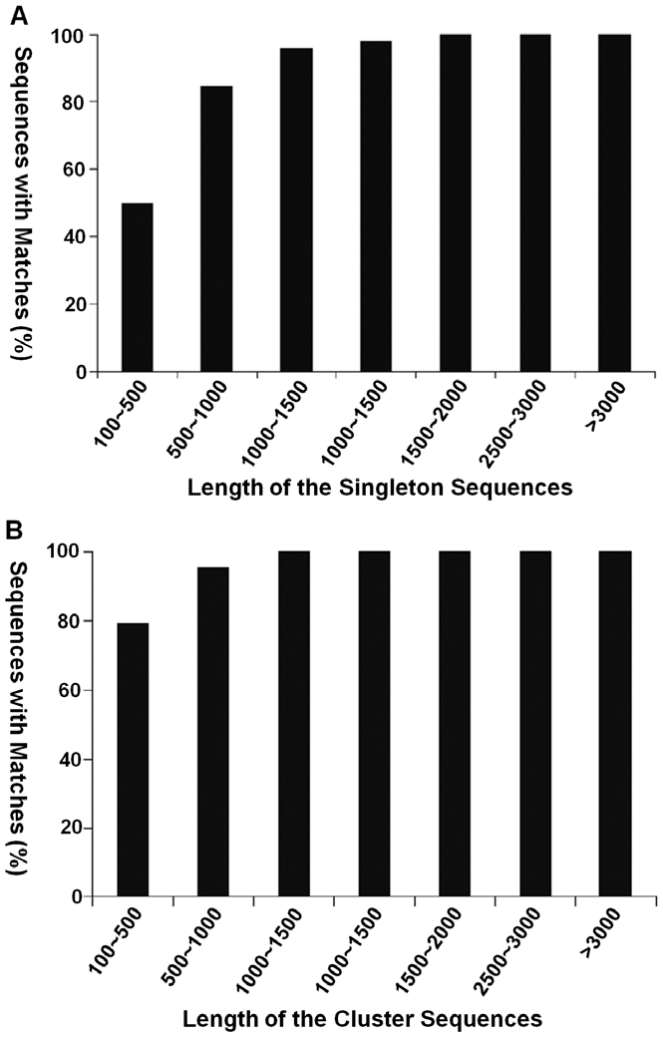
Effect of query length on the percentage of significant matches. (A) Singleton sequences. (B) Cluster sequences. The proportion of sequences with matches (with a cut-off E-value of 1.0E−5) in NR databases is greater among the longer assembled sequences.

For annotation, distinct gene sequences were first searched against the NCBI NR database using a cut-off E-value of 10^−5^. Although lack of genomic information in *Chenopodium* species, totally 61,698 genes (54.9% of all distinct sequences) including 3,272 clusters and 58,426 singletons showed significant matches (Table S1). As shown in Figure 2, the fraction of singletons that had BLAST matches was lower than for the clusters given their shorter length (mean size of 307 bp). For instance, a 100% of singletions longer than 2,000 bp achieved significant BLAST scores, and the proportion decreased sharply to 50.4% for sequences ranging from 100 to 500 bp (Figure 2A). However, clusters longer than only 1,000 bp totally achieved significant BLAST scores, and the proportion remained about 79.6% for sequences ranged in size from 100 bp to 500 bp (Figure 2B). Notably, a total of 49,970 genes (45.1%) were found no homologues in the current NCBI NR database, similar with the observations which have been previously made for several other transcriptomic studies [27]–[30]. These orphan sequences may represent novel genes in *C. amaranticolor* or are possibly derived from chimerical sequences (assemblage errors), the cDNA of untranslated regions or non-conserved regions of proteins.

**Figure 3:**
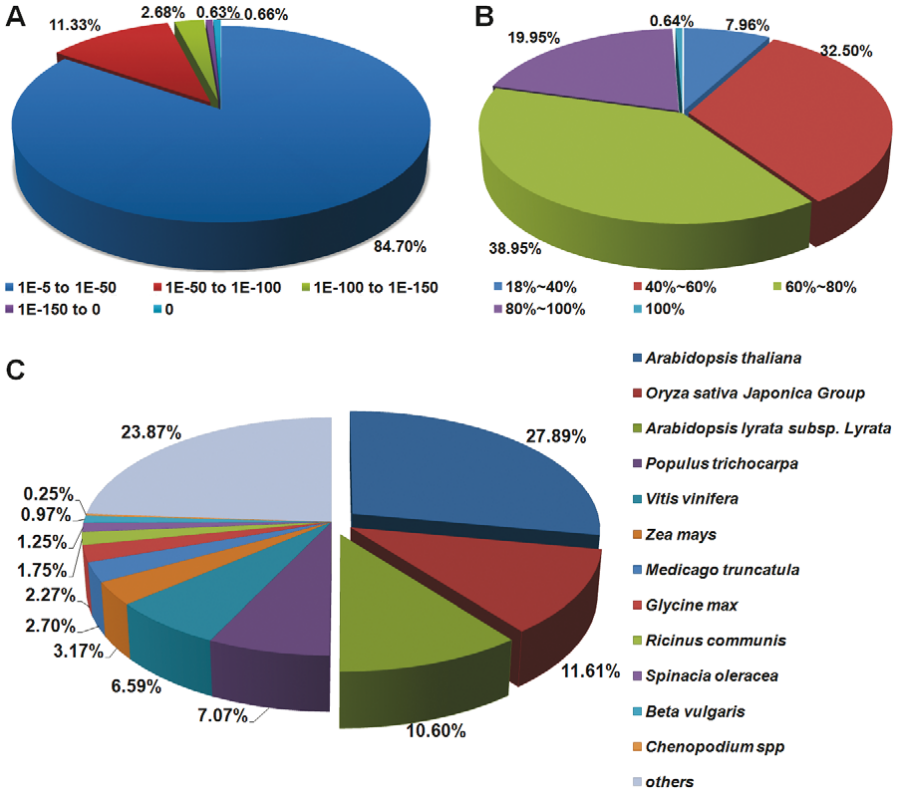
Characteristics of homology search of unigenes against the NR database. (A) E-value distribution of the top BLAST hits for each unigene with a cut-off E-value of 1.0E−5. (B) Similarity distribution of the best BLAST hits for each unigene. (C) Species distribution is shown as the percentage of the total homologous sequences with an E-value of at least 1.0E−5. We used all plant proteins in the NCBI NR database for homology search and extracted the first hit of each sequence for analysis.

The E-value distribution of the top hits in the NR database showed that 15.3% of the mapped sequences have strong homology (<1.0E^−50^), whereas 84.7% of the homolog sequences ranged between 1.0E^−5^ to 1.0E^−50^ (Figure 3A). The similarity distribution showed that 20.6% of the query sequences have a similarity higher than 80%, while 79.4% of the hits have a similarity ranging from 18% to 80% (Figure 3B). For species distribution, 27.9% of the distinct sequences have top matches (first hit) with sequences from the *Arabidopsis thaliana*, the genome of which has been sequenced in 2000 [31]. The next closest species were the *Oryza sativa* Japonica Group (11.6%), *A. lyrata* subsp. Lyrata (10.6%), *Populus trichocarpa* (7.1%), *Vitis vinifera* (6.6%) and *Zea mays* (3.2%) (Figure 3C). In addition, there were 772 and 152 distinct sequences with the highest homology to genes from *Spinacia oleracea* and *Chenopodium* spp., respectively. A total of 9,119 distinct sequences (14.8%) had the strongest similarity to genes of *O. sativa* Japonica Group and *Zea mays*, two types of monocot species, suggesting the interesting phylogenetic status of *C. amaranticolor*.

### GO, COG and KEGG classification

**Figure 4:**
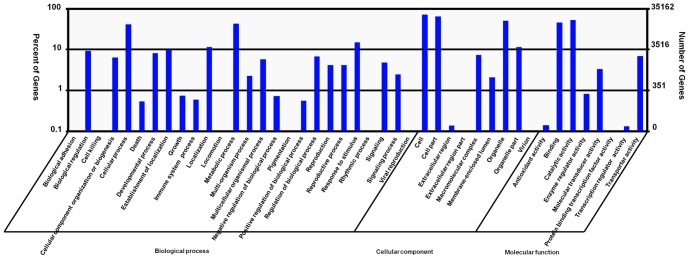
GO categories of biological process, cellular component and molecular function for the foliar transcriptome of *C. amaranticolor*. The right y-axis shows the number of genes in a category, while the left y-axis indicates the percentage of a specific category of genes in that main category.

Gene Ontology (GO) assignments were used to classify the functions of the predicted *C. amaranticolor* genes. Based on sequence homology, 35,162 sequences can be categorized into 43 functional groups (Figure 4). In each of the three main categories (biological process, cellular component and molecular function) of the GO classification, ‘Metabolic process’, “Cell” and “Catalytic activity” terms are dominant respectively. In agreement with GO assignments of other higher plant, such as *Taxus mairei* [32], *Camellia sinensis* [33], *Brassica juncea* [34] and *Siraitia grosvenorii* [35], we noticed a high-percentage of genes from categories of ‘Cellular process’, ‘Cell part’, ‘Organelle’ and ‘Binding’ (Figure 4), indicating that these GO terms are significant among all plant species and the mechanism of ‘Cellular process’ in different plants may be similar.

**Figure 5:**
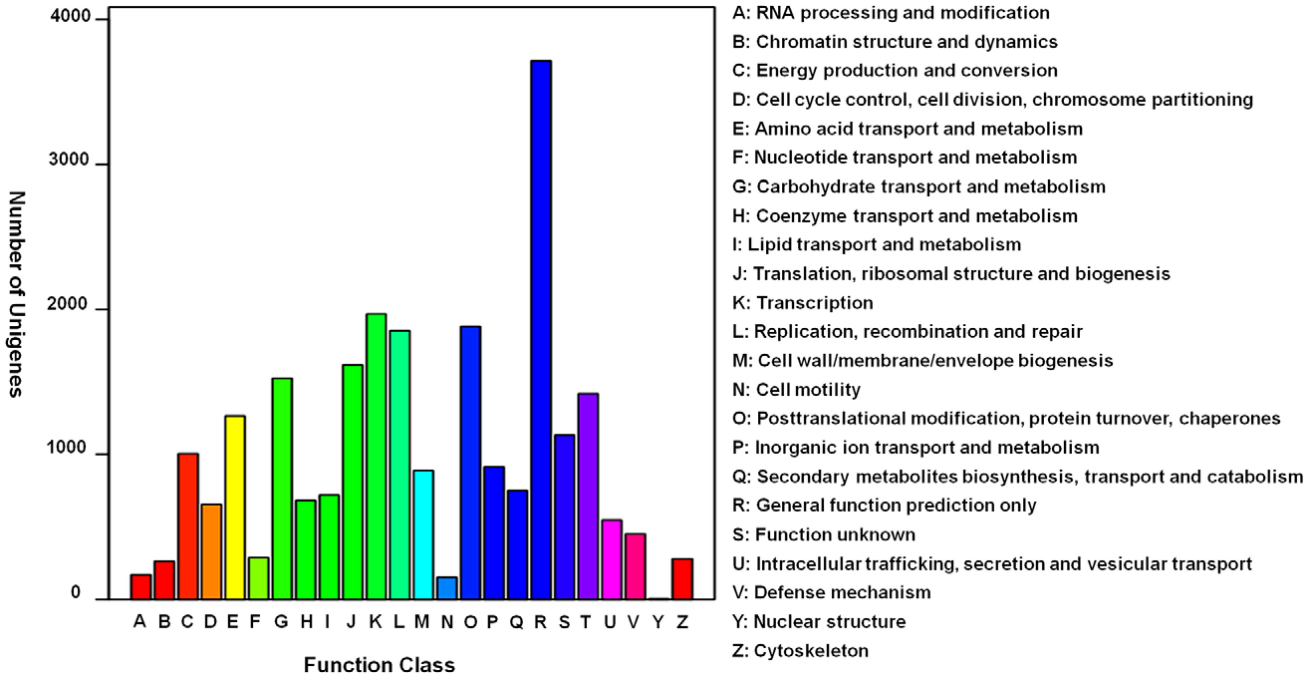
COG function classification of the foliar transcriptome of *C. amaranticolor*. Out of 62,482 nr hits, 24,147 sequences have a COG classification among the 24 categories.

To further evaluate the completeness of our transcriptome library and the effectiveness of our annotation process, we searched the annotated sequences for the genes involved in the clusters of orthologous group (COG) classifications. In total, out of 61,698 NR hits, 24,147 sequences have a COG classification (Figure 5). Among the 24 COG categories, the cluster for ‘General function prediction’ represents the largest group (3,714, 15.4%) followed by ‘Transcription’ (1,968, 8.2%) and ‘Replication, recombination and repair’ (1,855, 7.7%), while the category of nuclear structure (5, 0.02‰) represents the smallest group (Figure 5). It is noteworthy that 453 unigenes were assigned to the term of ‘Defense mechanism’.

To identify the biological pathways that are active in *C. amaranticolor*, we mapped the 61,698 annotated sequences to the reference canonical pathways in Kyoto Encyclopedia of Genes and Genomes (KEGG) [36]. In total, 27,042 sequences were assigned to 119 KEGG pathways. The pathways with most representation by the unique sequences were ‘Metabolic pathways’ (6,477, 23.95%), ‘Biosynthesis of secondary metabolites’ (3,795, 14.03%), and ‘Plant-pathogen interaction’ (2,346, 8.68%), providing a valuable genetic resource for the investigation of specific biological processes, functions and pathways in *C. amaranticolor*.

### Detection of resistance gene analogs and disease resistance signaling genes

To date, the majority of cloned and functional *R* genes described within the plant kingdom contain a conserved nucleotide binding site (NBS) and a C-terminal leucine-rich repeat (LRR) domain, known as NBS-LRRs [37], [38]. NBS-LRR-encoding genes represent one of the largest and most variable gene families in plants [39], with 149 members in genome of *A. thaliana* [40], 92 in *Brassica rapa* [41], 400–500 in *Medicago* [42], 464 and 483 in two genomes of *O. sativa* [43], 438 in potato [44], 416, 535 and 54 in three woody species poplar, grapevine and papaya, respectively [45], [46] (Yang et al., 2008; Porter et al., 2009). Herein, we compared the current transcriptome data with sequences from NCBI nucleotides and EST database to extract the unigenes represent candidate NBS-LRR *R* genes, and in total 738 unigenes were identified as resistance gene analogs (RGAs) (Table S2). In addition, a number of disease resistance signaling proteins, including NDR1, Rar1, Sgt1, EDS1, PAD4 and NPR1, which play key roles in the R proteins-directed signaling pathways [47], [48], were also identified from *C. amaranticolor* for the first time (Table S3). These overall findings establish a solid genetic basis for characterization of the *R* genes as well as the disease resistance signaling proteins in *C. amaranticolor*, and will certainly facilitate the future research of *Chenopodium* in *R* gene-mediated disease resistance.

### DGE library sequencing

An immediate application of our transcriptome sequence data includes analysis of gene expression variation over the stage of virus-induced HR in *C. amaranticolor* leaves. Five cDNA libraries derived from healthy leaves, TMV-6h, TMV-28h, CMV-6h, and CMV-20h were constructed and sequenced by using Illumina HiSeq™ 2000, respectively. After filtering the low quality reads, 12.5, 11.7, 12.2, 12.5, and 12.4 million clean reads were generated from the five samples, respectively (Table 2). To reveal the molecular events behind DGE profiles, these reads were mapped to the *C. amaranticolor* foliar transcriptome containing 112,453 unigenes. In total of 6,189,719, 5,826,515, 6,121,996, 6,339,408 and 5,948,757 reads derived from healthy leaves, TMV-6h, TMV-28h, CMV-6h and CMV-20h uniquely match with the unigenes in the reference database, respectively (Table 2). Reads mapped to a unique sequence are the most critical subset of the DGE libraries as they can explicitly identify a transcript. In this study, at least 93.93% of sequences in our transcriptome database could be unequivocally identified by the unique match reads (Table 2). To confirm if the number of detected genes increases proportionally to sequencing amount (total tag number), a saturation analysis was performed. As summarized in Figure S2, the results revealed a trend of saturation, in which the number of detected genes almost ceases to increase when the number of reads reaches ∼12 million. Next, the locations of DGE reads on reference genes were also evaluated [14], and the evenly distribution showed in Figure S3 indicated good randomness of these reads.

**Table 2:** Statistics of DGE sequencing data from *C. amaranticolor* infected with distinct viruses.

Sample	Total reads	Map to gene^a^		
		Reads mapping to gene^b^	Unique match reads^b^	Reads-mapped genes^b^
Healthy	12,503,742	6,232,961 (49.85%)	6,189,719 (49.50%)	106,816 (94.99%)
TMV-6h	11,664,230	5,866,379 (50.29%)	5,826,515 (49.95%)	105,624 (93.93%)
TMV-28h	12,204,006	6,165,075 (50.52%)	6,121,996 (50.16%)	107,101 (95.24%)
CMV-6h	12,501,382	6,383,164 (51.06%)	6,339,408 (50.71%)	105,648 (93.95%)
CMV-20h	12,389,583	5,993,814 (48.38%)	5,948,757 (48.01%)	108,457 (96.45%)

a The reads were mapped to foliar transcriptom of *C. amaranticolor* with 112, 452 unigenes.

b The conservative degree of mismatch was no more than 2 bp.

### Genes responding to virus inoculation in *C. amaranticolor*

**Figure 6:**
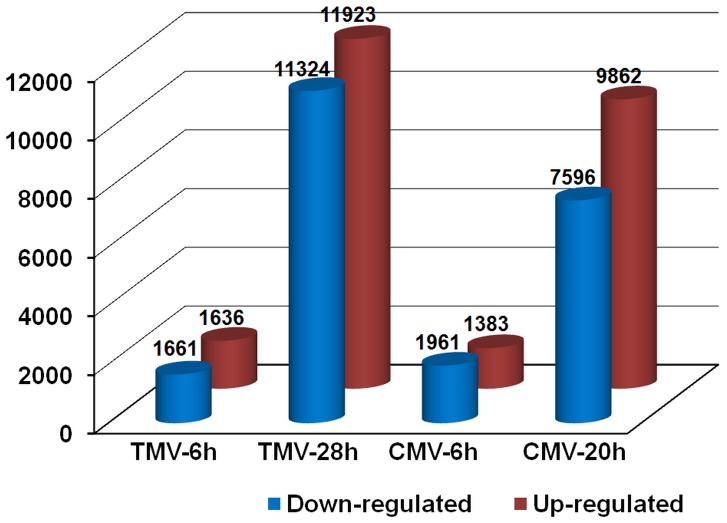
Changes in gene expression profiles of the TMV- and CMV-inoculated *C. amaranticolor* leaves at two different time points post-inoculation. The comparisons were made between inoculated and uninoculated (healthy leaves) at each time point. The numbers of up-regulated and down-regulated genes are summarized.

To identify genes showing a significant expression change in *C. amaranticolor* upon virus infection, the differentially expressed tags between virus-inoculated leaves (TMV-6h, CMV-6h, TMV-28h or CMV-20h) and healthy leaves were identified by an algorithm [49] based on the criteria of significance [False Discovery Rate (FDR)≤0.001 and |log_2_Ratio|≥1]. Accordingly, a total of 1,022,284 significantly changed tag entities were detected between healthy leaves and TMV-6h. Those tags were mapped to 3,297 genes with 1,636 genes up-regulated and 1,661 genes down-regulated, respectively, termed early-TMV-inducible genes (Figure 6 and Table S4). Between healthy leaves and TMV-28h, a total of 23,247 differentially expressed genes were detected with roughly the same amount of up-regulated genes (11,923) and down-regulated genes (11,324), and were named as late-TMV-inducible genes (Figure 6 and Table S5). Similarly, between healthy leaves and CMV-6h, 1,383 genes up-regulated and 1,961 genes down-regulated were defined as early-CMV-inducible genes (Figure 6 and Table S6), while between healthy and CMV-20h, a total of 17,458 differentially expressed genes, termed late-CMV-inducible genes, were identified with roughly the same amount of up-regulated genes (9,862) and down-regulated genes (7,596) (Figure 6 and Table S7). Collectively, these four datasets showed that numbers of both up-regulated (>2-fold) and down-regulated (<2-fold) genes increased following either TMV- or CMV-inoculation.

**Figure 7:**
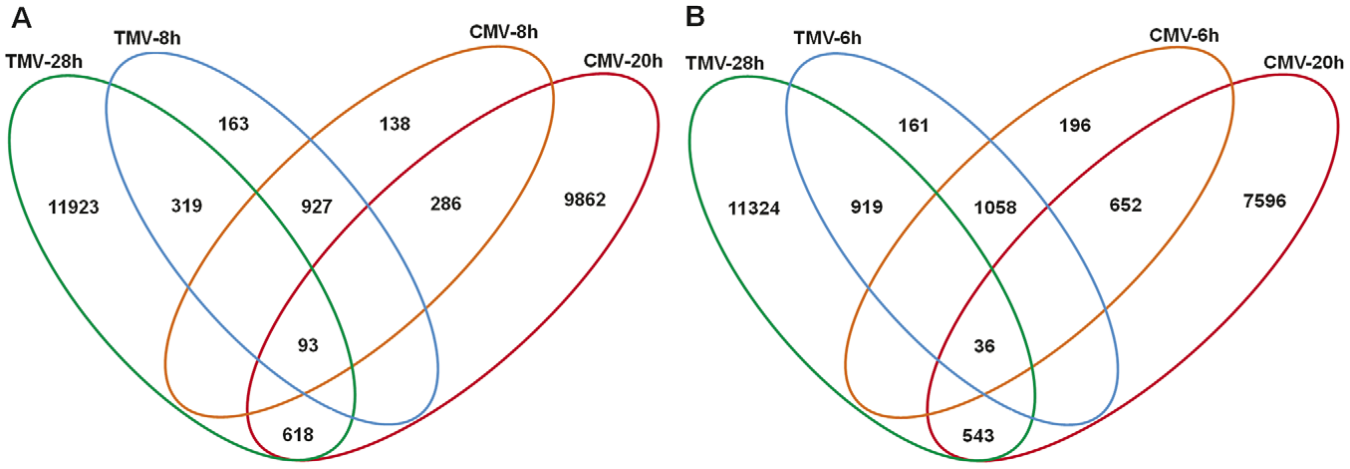
The generalized Venn diagrams with four datasets of TMV-6h, TMV-28h, CMV-6h and CMV-28h and their intersections. (A) Up-regulated genes and (B) down-regulated genes for each time point post-inoculation with TMV or CMV. All comparisons were made between inoculated and uninoculated (healthy) leaves at each time point.

Based on the four datasets, multiple comparisons were made to capture genes commonly regulated in *C. amaranticolor* upon different virus infection. As shown in the Venn diagrams (Figure 7A and 7B), a total of 1,238 unigenes including 319 up-regulated unigenes and 919 down-regulated unigenes were common between the early- and late-TMV-inducible genes (Table S8), while 286 up-regulated unigenes and 652 down-regulated unigenes overlapped between the early- and late-CMV-inducible genes (Table S9). Moreover, a total of 927 up-regulated unigenes and 1,058 down-regulated genes were common between the early-TMV- and early-CMV-inducible genes (Table S10), while between the late-TMV- and late-CMV-inducible genes, 6,181 up-regulated unigenes and 5,430 down-regulated genes, a total of 11,611 unigenes overlapped (Table S11). Further comparative analysis of the four datasets defined a core set of 461 unigenes comprising 93 up-regulated unigenes and 368 down-regulated unigenes which were commonly regulated by both TMV and CMV at all aforementioned post-inoculation times (Figure 7A and 7B and Table S12).

Efforts were also made to collect the alternatively regulated genes during the stage of HR induced by TMV or CMV. To this end, we identified that 275 unigenes were down-regulated in CMV-6h but up-regulated in CMV-20h, showing ‘down-up’ expression profiles, whereas 405 unigenes were regulated in the opposite direction (Table S13). For TMV infection, a total of 330 unigenes displayed ‘down-up’ expression profiles in TMV-6h/TMV-28h, while 194 unigenes showed ‘up-down’ expression profiles (Table S14). Further comparisons of these alternatively regulated genes identified 102 commonly ‘down-up’ regulated unigenes and 89 commonly ‘up-down’ regulated unigenes during both TMV and CMV infection (Table S15).

These overall datasets presented a significant number of genes commonly regulated in both TMV- and CMV-infected *C. amaranticolor*, suggesting the host plant might make use of some common pathways for defense against distinct viruses.

### Functional categorization of the identified genes

Of the identified differentially expressed genes, efforts have been first put forward to functional categorization of the 93 commonly up-regulated unigenes and 368 commonly down-regulated unigenes among the TMV-6h, TMV-28h, CMV-6h and CMV-20h. Apart from the unclassified ones, a total of 49 up-regulated unigenes and 111 down-regulated unigenes were categorized into various levels of GO biological processes, predominately in the ‘metabolism’ GO category including disaccharide, lipid, amino acids, protein, carbohydrate and primary metabolism, while the remaining ones were assigned to the GO processes of stress, defense, signal as well as transport (Table S12). Similarly, functional categorization of the 102 unigenes with ‘down-up’ expression profiles and 89 unigenes with ‘up-down’ profiles showed that, except the unclassified ones, the unigenes were also predominately categorized into the ‘metabolism’ GO category including protein and primary metabolism (Table S15).

To comprehensively assess the biological functions of the differentially expressed genes, all four sets of differentially expressed genes were mapped to KEGG database terms and compared with the whole transcriptome data, with a view to identify significantly enriched metabolic or signal transduction pathways. Among all the genes with KEGG pathway annotation, a total of 1,119, 1,174, 6,064 and 7,571 differentially expressed genes from the datasets of the early-TMV-inducible genes (TMV-6h), the late- TMV-inducible genes (TMV-28h), the early-CMV-inducible genes (CMV-6h) and the late-CMV-inducible genes (CMV-28h), were assigned to 97, 118, 102 and 118 KEGG pathways, respectively, suggesting the virus infection induces global alteration of the genes transcription profiles in *C. amaranticolor*.

Specific observations were made for the pathway of plant-pathogen interaction (PPI), in which, a total of 155, 141, 954 and 685 differentially expressed genes derived from TMV-6h, CMV-6h, TMV-28h and CMV-20h, respectively, were enriched. Interestingly, in TMV-6h and CMV-6h which represent the early stage of HR, over 80% of the differentially expressed genes in PPI pathway were significantly down-regulated, including RIN4, RPS2 and RPS5, the key proteins mediate effector-triggered HR cell death [5], [50]. Similarly, the host factors of EFR [51], [52] and WRKY29 [53], [54] in Mitogen-activated protein kinase (MAPK) signaling cascade, the well-known defense marker PR1 [55] and the essential gene *COI1* for jasmonate-regulated defense [56] were all down-regulated at the early stage. In contrast, at the late stage of HR (CMV-20h and TMV-28h), the majority (>80%) of the differentially expressed genes enriched in PPI were highly up-regulated, such as RIN4, RPS5 and the MAPK signaling cascade-associated genes MKK4/5 [57] and WRKY25/33 [58]–[60]. It is noteworthy to mention the ‘down-up’ expression profiles of the *RIN4* and *RPS5*, which suggest essential roles of these two genes during virus-induced HR and merit further investigation. In addition, a particular interest was given to NHO1, a non-host resistance gene [61], [62], which is the unique gene consistently up-regulated in all four samples, indicating its yet undetermined role in virus resistance. Collectively, these observations defined that number of unigenes in PPI pathway underwent complicated regulation mechanisms during virus-induced HR, leading to a first dynamic picture of the changes in the overall pattern of gene expression in PPI pathway of *C. amaranticolor*.

### Conclusions

This study represents the first application of Illumina sequencing technology for genomic studies in *C. amaranticolor*, a host with the broad-spectrum virus resistance. A single run produced 112,453 unigenes with 62,482 sequences having an above cut-off BLAST result. From this foliar transcriptome database, 738 RGAs and a number of sequences represent disease resistance signaling proteins were identified. By using Illumina sequencing-based DGE system, we further analyzed the gene expression profiles during virus-induced HR in *C. amaranticolor* leaves, and identified numbers of candidate genes specifically and commonly regulated by TMV and CMV at early and late stages of the HR. This genome-scale transcriptional information provides a substantial contribution to the sequence resources for *C. amaranticolor* and will particularly aid in understanding the genetic mechanism of the broad-spectrum virus resistance conferred by this host.

## Materials and Methods

### Plant materials preparation

Leaves of 6∼7 week-old *C. amaranticolor* grown in the greenhouse were inoculated with CMV SD strain [63] and TMV U1 [64], respectively. Briefly, infectious sap was prepared with one gram of fresh TMV- or CMV-infected *Nicotiana tabacum* leaves that were macerated in 10 mL of inoculation buffer (50 mM KH_2_P0_4_, pH 7.0, 1% Celite), and was mechanically inoculated onto *C. amaranticolor* leaves dusted with carborundum powder (600 mesh). The virus-inoculated leaf tissues were harvested using half leaf method as follows. Half of each virus-inoculated leaf was first sampled at 6 hpi, while the remaining halves were collected at 20 hpi for CMV-inoculated leaves and at 28 hpi for TMV-inoculated leaves, at which time point local lesions became just visible. These samples were accordingly named as TMV-6h, CMV-6h, TMV-28h and CMV-20h. Notably, the leaf tissues of CMV-6h or TMV-6h were selected only when the remaining halves showed significant number of local lesions at 20 hpi (CMV) or 28 hpi (TMV), in order to ensure the tissues of CMV-6h and TMV-6h being fully infected. Leaves of the healthy plants were also harvested as control. All samples were immediately frozen in liquid nitrogen and were stored at −80°C until use.

### cDNA library preparation for transcriptome analysis

Total RNA was extracted using TRIzol® reagent (Invitrogen) according to the manufacturer's protocol. RNA integrity was confirmed with RNA6000 Nano Assay using the 2100 Bioanalyzer (Agilent Technologies, Palo Alto, CA). The samples for transcriptome analysis were prepared using Illumina's kit following manufacturer's instruction. Briefly, using oligo (dT) magnetic beads (Invitrogen), poly(A) mRNA was first isolated from 6 µg of total RNA, which was extracted from the mixed materials containing equal amount of healthy leaves, TMV-8h, CMV-8h, TMV-28h, and CMV-20h. The mRNA was then fragmented into smaller pieces at 70°C for 5 min in the fragmentation buffer (Ambion). Taking these short mRNA fragments as templates, reverse transcriptase and random hexamer-primer were used to synthesize the first-strand cDNA. This was followed by second strand cDNA synthesis using DNA polymerase I (Invitrogen) and RNaseH (Invitrogen). The resulted short cDNA fragments were purified with QiaQuick PCR extraction kit (Qiagen) followed by end repair, adding poly(A), and ligation of sequencing adaptors with Illumina's adaptor oligo mix. The fragments were purified for the section of approximate 200 bp long using Qiaquick Gel Extraction Kit (Qiagen) and were further enriched with PCR to create the final sequencing cDNA library.

### Analysis of Illumina sequencing results

The cDNA library was sequenced from both of the 5′ and 3′ ends on the Illumina HiSeq™ 2000 platform. The fluorescent images deconvolution and quality value calculation were performed using the Illumina data processing pipeline (version 1.6), in which 90 bp paired-end reads were obtained. Before assembly, the raw reads were filtered to obtain high-quality clean reads by removing low quality reads as well as reads with adaptor sequences or containing unknown nucleotides larger than 5%.

*De novo* assembly of the clean reads was performed using SOAPdenovo software [65]. Briefly, SOAPdenovo first combines the reads with certain length of overlap to form contigs, and the contigs from the same transcript as well as the distances between these contigs were further detected by using paired-end reads. Next, SOAPdenovo connects the contigs using N to represent unknown sequences between each two contigs, resulting in Scaffolds. Finally, paired-end reads are used again for gap filling of scaffolds to generate Unigenes. To determine the sequence direction, the unigenes were aligned by blastx (E-value<0.00001) to protein databases in a priority order of NCBI NR, Swiss-Prot, KEGG and COG. Once a unigene is aligned to none of the above databases, ESTScan [66] is introduced to decide its sequence direction. In this study, we provide the unigenes with sequence directions from 5′ end to 3′ end, while those without any direction were from assembly software.

The generated unigenes were used for blast search and annotation against the plant protein database of NR with a significant threshold of E-value≤10^−5^. Functional categorization by GO terms [67] was performed using Blast2GO software [68] with E-value cut-off at 10^−5^. The COG and KEGG pathways annotation was carried out using Blastall software (E-value threshold of 10^−5^) against the COG database [69] and the KEGG database [36], respectively.

### Digital gene expression library preparation and sequencing

Tag library preparation for the distinct *C. amaranticolor* sample (healthy leaves, TMV-8h, TMV-28h, CMV-8h and CMV-20h) was performed in parallel using Illumina gene expression sample preparation kit as described in cDNA library preparation. The generated cDNA library was sequenced using Illumina HiSeq™ 2000, resulting in the raw image data, which, subsequently, was transformed by base calling into sequence data.

### Digital gene expression tags analysis

Prior to mapping reads to the reference database, all the raw reads were filtered to remove low quality reads as well as reads with adaptor sequences or containing unknown nucleotides larger than 5%. The filtered sequence data were named as clean reads, on which all following analyses were based. For annotation, all reads were mapped to the reference sequences using SOAPaligner/soap2 [65] with a maximum of no more than 2 nucleotides mismatches. The reads mapped to reference sequences from multiple genes were filtered, while the remaining reads were designed as unambiguous tags. For gene expression analysis, the number of expressed reads was counted and normalized using RPKM (reads per kb per million reads) [66].

A statistical analysis of the frequency of each read in the different cDNA libraries was further performed to screen the differentially expressed genes in *C. amaranticolor* upon virus infection. Statistical comparison was performed with a strict algorithm referring to the method described previously [49]. FDR was used to determine the threshold of P value in multiple test and analysis. The threshold as “FDR≤0.001” was introduced to judge the significance of gene expression difference. The identified genes with differentially expression levels were then mapped to terms in GO and KEGG database, looking for the significantly enriched KEGG terms comparing to the genome background.

### Data deposition

The nucleotide sequences of raw reads from this study were submitted to NCBI Gene Expression Omnibus under the accession number GSE38451.

## Supporting Information

Figure S1 **Summary of the** ***C. amaranticolor*** **transcriptomic sequences.** (A) Size distribution of Illumina sequencing contigs. (B) Size distribution of distinct sequences after paired-end and gap filling.
(TIF)

Figure S2 **Relationship between the number of identified genes and the sequencing amount (total read number).** The figures represent DGE reads of healthy leaves (A), TMV-6h (B), TMV-28h (C), CMV-6h (D) and CMV-20h (E), respectively, showing a trend of saturation. When the sequencing amount reaches 12 millions, the number of identified genes almost ceases to increase.
(TIF)

Figure S3 Distribution of DGE reads derived from **healthy leaves (A), TMV-6h (B), TMV-28h (C), CMV-6h (D) and CMV-20h (E)** on reference genes in **the** ***C. amaranticolor*** **transcriptome.**
(TIF)

Table S1 **Top BLAST hits from NCBI nr database. BLAST results against the NCBI nr database for all the clusters and singletons with a cut-off E value above 10^−5^ are shown.**
(RAR)

Table S2 **Putative resistance gene analogs (RGAs) identified from the foliar transcriptome of** ***C. amaranticolor*** **.**
(XLS)

Table S3 **Putative disease resistance signaling proteins identified from the foliar transcriptome of** ***C. amaranticolor***.
(XLS)

Table S4 **Differentially expressed Genes between TMV-6h and healthy leaves (early-TMV-inducible genes).** RPKM: reads per kb per million reads. Raw intensity: the total number of reads sequenced for each gene. FDR: false discovery rate. The significance of gene expression difference between samples was identified by using FDR<0.001 and the absolute value of log2Ratio ≤1 as the threshold. To calculate the log2Ratio and FDR, we set RPKM value of 0.001 instead of 0 for genes that do not express in one sample.
(XLS)

Table S5 **Differentially expressed genes between TMV-28h and healthy leaves (late-TMV-inducible genes).** RPKM: reads per kb per million reads. Raw intensity: the total number of reads sequenced for each gene. FDR: false discovery rate. The significance of gene expression difference between samples was identified by using FDR<0.001 and the absolute value of log2Ratio ≤1 as the threshold. To calculate the log2Ratio and FDR, we set RPKM value of 0.001 instead of 0 for genes that do not express in one sample.
(RAR)

Table S6 **Differentially expressed genes between CMV-6h and healthy leaves (early-CMV-inducible genes).** RPKM: reads per kb per million reads. Raw intensity: the total number of reads sequenced for each gene. FDR: false discovery rate. The significance of gene expression difference between samples was identified by using FDR<0.001 and the absolute value of log2Ratio ≤1 as the threshold. To calculate the log2Ratio and FDR, we set RPKM value of 0.001 instead of 0 for genes that do not express in one sample.
(XLS)

Table S7 **Differentially expressed genes between CMV-20h and healthy leaves (late-CMV-inducible genes).** RPKM: reads per kb per million reads. Raw intensity: the total number of reads sequenced for each gene. FDR: false discovery rate. The significance of gene expression difference between samples was identified by using FDR<0.001 and the absolute value of log2Ratio ≤1 as the threshold. To calculate the log2Ratio and FDR, we set RPKM value of 0.001 instead of 0 for genes that do not express in one sample.
(RAR)

Table S8 **Common genes between the early-TMV-inducible genes and the late-TMV-inducible genes.**
(XLS)

Table S9 **Common genes between the early-CMV-inducible genes and the late-CMV-inducible genes.**
(XLS)

Table S10 **Common genes between the early-TMV-inducible genes and the early-CMV-inducible genes.**
(XLS)

Table S11 **Common genes between the late-TMV-inducible genes and the late-CMV-inducible genes.**
(RAR)

Table S12 **Common genes among the early-TMV-inducible genes, the early-CMV-inducible genes, the late-TMV-inducible genes and the late-CMV-inducible genes.**
(XLS)

Table S13 **Genes showed ‘down-up’ or ‘up-down’ expression profiles during the TMV-induced HR.**
(XLS)

Table S14 **Genes showed ‘down-up’ or ‘up-down’ expression profiles during the CMV-induced HR.**
(XLS)

Table S15 **Genes showed ‘down-up’ or ‘up-down’ expression profiles during both TMV- and CMV-induced HR.**
(XLS)

## References

[1] Heath MC (2000) Hypersensitive responses-related death. Plant Mol Biol 44: 321–334. 11199391 10.1023/a:1026592509060

[2] Shirasu K, Schulze-Lefert P (2000) Regulators of cell death in disease resistance. Plant Mol Biol 44: 371–385. 11199395 10.1023/a:1026552827716

[3] Flor HH (1971) Current status of gene-for-gene concept. Annu Rev Phytopathol 9: 275–296.

[4] Dangl JL, Jones JD (2001) Plant pathogens and integrated defence responses to infection. Nature 411: 826–833. 11459065 10.1038/35081161

[5] Jones JD, Dangl JL (2006) The plant immune system. Nature 444: 323–329. 17108957 10.1038/nature05286

[6] Baker B, Zambryski P, Staskawicz B, Dinesh-Kumar SP (1997) Signaling in plant-microbe interactions. Science 276: 726–733. 9115193 10.1126/science.276.5313.726

[7] Durrant WE, Dong X (2004) Systemic acquired resistance. Annu Rev Phytopathol 42: 185–209. 15283665 10.1146/annurev.phyto.42.040803.140421

[8] Spoel SH, Dong X (2012) How do plants achieve immunity? Defence without specialized immune cells. Nat Rev Immunol 12: 89–100. 22273771 10.1038/nri3141

[9] CMI/AAB (1984) Descriptions of Plant Viruses, July Commonwealth Mycological Institute and the Association of Applied Biologists. Unwin Brothers Ltd. Old Woking Surrey.

[10] Cooper B (2001) Collateral gene expression changes induced by distinct plant viruses during the hypersensitive resistance reaction in *Chenopodium amaranticolor* . Plant J 26: 339–349. 11439122 10.1046/j.1365-313x.2001.01030.x

[11] Visedo G, Fernandez-Piqueras J, Garcia JA (1990) Isozyme profiles associated with the hypersensitive response of Chenopodium foetidum to plum pox virus infection. Physiologia Plantarum 78: 218–224.

[12] Schmitz I, Rao AL (1996) Molecular studies on bromo virus capsid protein I. Characterization of cell-to-cell movementdefective RNA3 variants of *Brome mosaic virus* . Virology 226: 281–293. 8955048 10.1006/viro.1996.0656

[13] Canto T, Palukaitis P (1999) The hypersensitive response to cucumber mosaic virus in Chenopodium amaranticolor requires virus movement outside the initially infected cell. Virology 265: 74–82. 10603319 10.1006/viro.1999.0028

[14] Wang Z, Gerstein M, Snyder M (2009) RNA-Seq: a revolutionary tool for transcriptomics. Nat Rev Genet 10: 57–63. 19015660 10.1038/nrg2484 PMC2949280

[15] Ward JA, Ponnala L, Weber CA (2012) Strategies for transcriptome analysis in nonmodel plants. Am J Bot 99: 267–276. 22301897 10.3732/ajb.1100334

[16] Yang SS, Tu ZJ, Cheung F, Xu WW, Lamb JF, et al (2011) Using RNA-Seq for gene identification, polymorphism detection and transcript profiling in two alfalfa genotypes with divergent cell wall composition in stems. BMC Genomics 12: 199. 21504589 10.1186/1471-2164-12-199 PMC3112146

[17] Wang W, Wang Y, Zhang Q, Qi Y, Guo D (2009) Global characterization of *Artemisia annua* glandular trichome transcriptome using 454 pyrosequencing. BMC Genomics 10: 465. 19818120 10.1186/1471-2164-10-465 PMC2763888

[18] Logacheva MD, Kasianov AS, Vinogradov DV, Samigullin TH, Gelfand MS, et al (2011) *De novo* sequencing and characterization of floral transcriptome in two species of buckwheat (*Fagopyrum*). BMC Genomics 12: 30. 21232141 10.1186/1471-2164-12-30 PMC3027159

[19] Wall PK, Leebens-Mack J, Chanderbali AS, Barakat A, Wolcott E, et al (2009) Comparison of next generation sequencing technologies for transcriptome characterization. BMC Genomics 10: 347. 19646272 10.1186/1471-2164-10-347 PMC2907694

[20] Novaes E, Drost DR, Farmerie WG, Pappas GJ Jr, Grattapaglia D, et al (2008) High-throughput gene and SNP discovery in *Eucalyptus grandis*, an uncharacterized genome. BMC Genomics 9: 312. 18590545 10.1186/1471-2164-9-312 PMC2483731

[21] Bellin D, Ferrarini A, Chimento A, Kaiser O, Levenkova N, et al (2009) Combining next-generation pyrosequencing with microarray for large scale expression analysis in non-model species. BMC Genomics 10: 555. 19930683 10.1186/1471-2164-10-555 PMC2790472

[22] Su CL, Chao YT, Alex Chang YC, Chen WC, Chen CY, et al (2011) *De novo* assembly of expressed transcripts and global analysis of the *Phalaenopsis aphrodite* transcriptome. Plant Cell Physiol 52: 1501–1514. 21771864 10.1093/pcp/pcr097

[23] Collins LJ, Biggs PJ, Voelckel C, Joly S (2008) An approach to transcriptome analysis of non-model organisms using short-read sequences. Genome Informatics 21: 3–14. 19425143

[24] Scholthof KB, Adkins S, Czosnek H, Palukaitis P, Jacquot E, et al (2011) Top 10 plant viruses in molecular plant pathology. Mol Plant Pathol 2011: 938–954. 10.1111/j.1364-3703.2011.00752.x PMC6640423 22017770

[25] Li R, Zhu H, Ruan J, Qian W, Fang X, et al (2010) *De novo* assembly of human genomes with massively parallel short read sequencing. Genome Res 20: 265–272. 20019144 10.1101/gr.097261.109 PMC2813482

[26] Pertea G, Huang X, Liang F, Antonescu V, Sultana R, et al (2003) TIGR Gene Indices clustering tools (TGICL): a software system for fast clustering of large EST datasets. Bioinformatics 19: 651–652. 12651724 10.1093/bioinformatics/btg034

[27] Mittapalli O, Bai X, Mamidala P, Rajarapu SP, Bonello P, et al (2010) Tissue specific transcriptomics of the exotic invasive insect pest emerald ash borer. PLoS ONE 5: e13708. 21060843 10.1371/journal.pone.0013708 PMC2965670

[28] Liang H, Carlson JE, Leebens-Mack JH, Wall PK, Mueller LA, et al (2008) An EST database for *Liriodendron tulipifera* L. floral buds: the first EST resource for functional and comparative genomics in *Liriodendron* . Tree Genet Genom 4: 419–433.

[29] Bai X, Mamidala P, Rajarapu SP, Jones SC, Mittapalli O (2011) Transcriptomics of the Bed Bug (*Cimex lectularius*). PLoS ONE 6: e16336. 21283830 10.1371/journal.pone.0016336 PMC3023805

[30] Wang XW, Luan JB, Li JM, Bao YY, Zhang CX, et al (2010) *De novo* characterization of a whitefly transcriptome and analysis of its gene expression during development. BMC Genomics 11: 400. 20573269 10.1186/1471-2164-11-400 PMC2898760

[31] The Arabidopsis Genome Initiative (2000) Analysis of the genome sequence of the flowering plant *Arabidopsis thaliana* . Nature 408: 796–815. 11130711 10.1038/35048692

[32] Hao DC, Ge G, Xiao P, Zhang Y, Yang L (2011) The first insight into the tissue specific *Taxus* transcriptome via Illumina second generation sequencing. PLoS ONE 6: e21220. 21731678 10.1371/journal.pone.0021220 PMC3120849

[33] Shi CY, Yang H, Wei CL, Yu O, Zhang ZZ, et al (2011) Deep sequencing of the Camellia sinensis transcriptome revealed candidate genes for major metabolic pathways of tea-specific compounds. BMC Genomics 12: 131. 21356090 10.1186/1471-2164-12-131 PMC3056800

[34] Sun Q, Zhou GF, Cai YF, Fan YH, Zhu XY, et al (2012) Transcriptome analysis of stem development in the tumourous stem mustard Brassica juncea var. tumida Tsen et Lee by RNA sequencing. BMC Plant Biol 12: 53. 22520079 10.1186/1471-2229-12-53 PMC3349559

[35] Tang Q, Ma XJ, Mo CM, Wilson IW, Song C, et al (2011) An efficient approach to finding Siraitia grosvenorii triterpene biosynthetic genes by RNA-seq and digital gene expression analysis. BMC Genomics 12: 343. 21729270 10.1186/1471-2164-12-343 PMC3161973

[36] Kanehisa M, Araki M, Goto S, Hattori M, Hirakawa M, et al (2008) KEGG for linking genomes to life and the environment. Nucleic Acids Res. 36: D480–D484. 10.1093/nar/gkm882 PMC2238879 18077471

[37] van der Biezen EA, Jones JD (1998) Plant disease-resistance proteins and the gene-for-gene concept. Trends Biochem Sci 23: 454–456. 9868361 10.1016/s0968-0004(98)01311-5

[38] Lukasik E, Takken FL (2009) STANDing strong, resistance proteins instigators of plant defence. Curr Opin Plant Biol 12: 427–436. 19394891 10.1016/j.pbi.2009.03.001

[39] Clark RM, Schweikert G, Toomajian C, Ossowski S, Zeller G, et al (2007) Common sequence polymorphisms shaping genetic diversity in *Arabidopsis thaliana* . Science 317: 338–342. 17641193 10.1126/science.1138632

[40] Meyers BC, Kozik A, Griego A, Kuang H, Michelmore RW (2003) Genome-wide analysis of NBS-LRR-encoding genes in Arabidopsis. Plant Cell 15: 809–834. 12671079 10.1105/tpc.009308 PMC152331

[41] Mun JH, Yu HJ, Park S, Park BS (2009) Genome-wide identification of NBS-encoding resistance genes in *Brassica rapa*. Mol. Genet. Genomics 282: 617–631. 10.1007/s00438-009-0492-0 PMC2777221 19838736

[42] Ameline-Torregrosa C, Wang BB, O'Bleness MS, Deshpande S, Zhu H, et al (2008) Identification and characterization of nucleotide-binding site-leucine-rich repeat genes in the model plant *Medicago truncatula* . Plant Physiol 146: 5–21. 17981990 10.1104/pp.107.104588 PMC2230567

[43] Yang S, Feng Z, Zhang X, Jiang K, Jin X, et al (2006) Genome wide investigation on the genetic variations of rice disease resistance genes. Plant Mol Biol 62: 181–193. 16915523 10.1007/s11103-006-9012-3

[44] Jupe F, Pritchard L, Etherington GJ, MacKenzie K, Cock PJA, et al (2012) Identification and localisation of the NB-LRR gene family within the potato genome. BMC Genomics 13: 75. 22336098 10.1186/1471-2164-13-75 PMC3297505

[45] Yang S, Zhang X, Yue JX, Tian D, Chen JQ (2008) Recent duplications dominate NBS-encoding gene expansion in two woody species. Mol Genet Genomics 280: 187–198. 18563445 10.1007/s00438-008-0355-0

[46] Porter BW, Paidi M, Ming R, Alam M, Nishijima WT, et al (2009) Genome-wide analysis of *Carica papaya* reveals a small NBS resistance gene family. Mol Genet Genomics 281: 609–626. 19263082 10.1007/s00438-009-0434-x

[47] Mur LA, Kenton P, Lloyd AJ, Ougham H, Prats E (2008) The hypersensitive response; the centenary is upon us but how much do we know? J Exp Bot 59: 501–520. 18079135 10.1093/jxb/erm239

[48] Caldwell KS, Michelmore RW (2009) *Arabidopsis thaliana* genes encoding defense signaling and recognition proteins exhibit contrasting evolutionary dynamics. Genetics 181: 671–684. 19064707 10.1534/genetics.108.097279 PMC2644955

[49] Audic S, Claverie JM (1997) The significance of digital gene expression profiles. Genome Res 7: 986–995. 9331369 10.1101/gr.7.10.986

[50] Dodds PN, Rathjen JP (2010) Plant immunity: Towards an integrated view of plant-pathogen interactions. Nat Rev Genet 11: 539–548. 20585331 10.1038/nrg2812

[51] Zipfel C, Kunze G, Chinchilla D, Caniard A, Jones JDG, et al (2006) Perception of the bacterial PAMP EF-Tu by the receptor EFR restricts Agrobacterium-mediated transformation. Cell 125: 749–760. 16713565 10.1016/j.cell.2006.03.037

[52] Nicaise V, Roux M, Zipfel C (2009) Recent advances in PAMP-triggered immunity against bacteria: pattern recognition receptors watch over and raise the alarm. Plant Physiol 150: 1638–1647. 19561123 10.1104/pp.109.139709 PMC2719144

[53] Asai T, Tena G, Plotnikova J, Willmann MR, Chiu W-L, et al (2002) MAP kinase signalling cascade in Arabidopsis innate immunity. Nature 415: 977–983. 11875555 10.1038/415977a

[54] Pandey SP, Somssich IE (2009) The role of WRKY transcription factors in plant immunity. Plant Physiol 150: 1648–1655. 19420325 10.1104/pp.109.138990 PMC2719123

[55] van Loon IC, van Strein EA (1999) The families of pathogenesis-related proteins, their activities, and comparative analysis of PR-1 type proteins. Physiol Mol Plant Pathol 55: 85–97.

[56] Xie D, Feys BF, James S, Nieto-Rostro M, Turner JG (1998) COI1: an Arabidopsis gene required for jasmonate-regulated defense and fertility. Science 280: 1091–1094. 9582125 10.1126/science.280.5366.1091

[57] Pitzschke A, Schikora A, Hirt H (2009) MAPK cascade signalling networks in plant defence. Curr Opin Plant Biol 12: 1–6. 19608449 10.1016/j.pbi.2009.06.008

[58] Zheng Z, Mosher SL, Fan B, Klessig DF, Chen Z (2007) Functional analysis of Arabidopsis WRKY25 transcription factor in plant defense against Pseudomonas syringae. BMC Plant Biol 7: 2. 17214894 10.1186/1471-2229-7-2 PMC1780049

[59] Zheng Z, Qamar SA, Chen Z, Mengiste T (2006) Arabidopsis WRKY33 transcription factor is required for resistance to necrotrophic fungal pathogens. Plant J 48: 592–605. 17059405 10.1111/j.1365-313X.2006.02901.x

[60] Mao G, Meng X, Liu Y, Zheng Z, Chen Z, et al (2011) Phosphorylation of a WRKY transcription factor by two pathogenresponsive MAPKs drives phytoalexin biosynthesis in Arabidopsis. Plant Cell 23: 1639–1653. 21498677 10.1105/tpc.111.084996 PMC3101563

[61] Lu M, Tang X, Zhou JM (2001) Arabidopsis NHO1 is required for general resistance against *Pseudomonas bacteria* . Plant Cell 13: 437–447. 11226196 10.1105/tpc.13.2.437 PMC102253

[62] Kang L, Li J, Zhao T, Xiao F, Tang X, et al (2003) Interplay of the Arabidopsis nonhost resistance gene NHO1 with bacterial virulence. Proc Natl Acad Sci USA 100: 3519–3524. 12626746 10.1073/pnas.0637377100 PMC152325

[63] Hou WN, Duan CG, Fang RX, Zhou XY, Guo HS (2011) Satellite RNA reduces expression of the 2b suppressor protein resulting in the attenuation of symptoms caused by *Cucumber mosaic virus* infection. Mol Plant Pathol 12: 595–605. 21722297 10.1111/j.1364-3703.2010.00696.x PMC6640352

[64] Dawson WO, Beck DL, Knorr DA, Grantham GL (1986) cDNA cloning of the complete genome of *tobacco mosaic virus* and production of infectious transcripts. Proc Natl Acad Sci USA 83: 1832–1836. 16593669 10.1073/pnas.83.6.1832 PMC323178

[65] Li R, Yu C, Li Y, Lam TW, Yiu SM, et al (2009) SOAP2: An improved ultrafast tool for short read alignment. Bioinformatics 25: 1966–1967. 19497933 10.1093/bioinformatics/btp336

[66] Mortazavi A, Williams BA, McCue K, Schaeffer L, Wold B (2008) Mapping and quantifying mammalian transcriptomes by RNA-Seq. Nature Methods 5: 621–628. 18516045 10.1038/nmeth.1226 PMC13303166

[67] Ashburner M, Ball CA, Blake JA, Botstein D, Butler H, et al (2000) Gene Ontology: tool for the unification of biology. Nat Genet 25: 25–29. 10802651 10.1038/75556 PMC3037419

[68] Conesa A, Gotz S, Garcia-Gomez JM, Terol J, Talon M, et al (2005) Blast2GO: a universal tool for annotation, visualization and analysis in functional genomics research. Bioinformatics 21: 3674–3676. 16081474 10.1093/bioinformatics/bti610

[69] Tatusov RL, Natale DA, Garkavtsev IV, Tatusova TA, Shankavaram UT, et al (2001) The COG database: new developments in phylogenetic classification of proteins from complete genomes. Nucleic Acids Res 29: 22–28. 11125040 10.1093/nar/29.1.22 PMC29819

