# Supplementary figures and images for: De Novo Foliar Transcriptome of Chenopodium amaranticolor and Analysis of Its Gene Expression During Virus-Induced Hypersensitive Response
Source: PLoS One. 2012 Sep 28;7(9):e45953. doi: 10.1371/journal.pone.0045953 (PMC3461033; doi:10.1371/journal.pone.0045953)

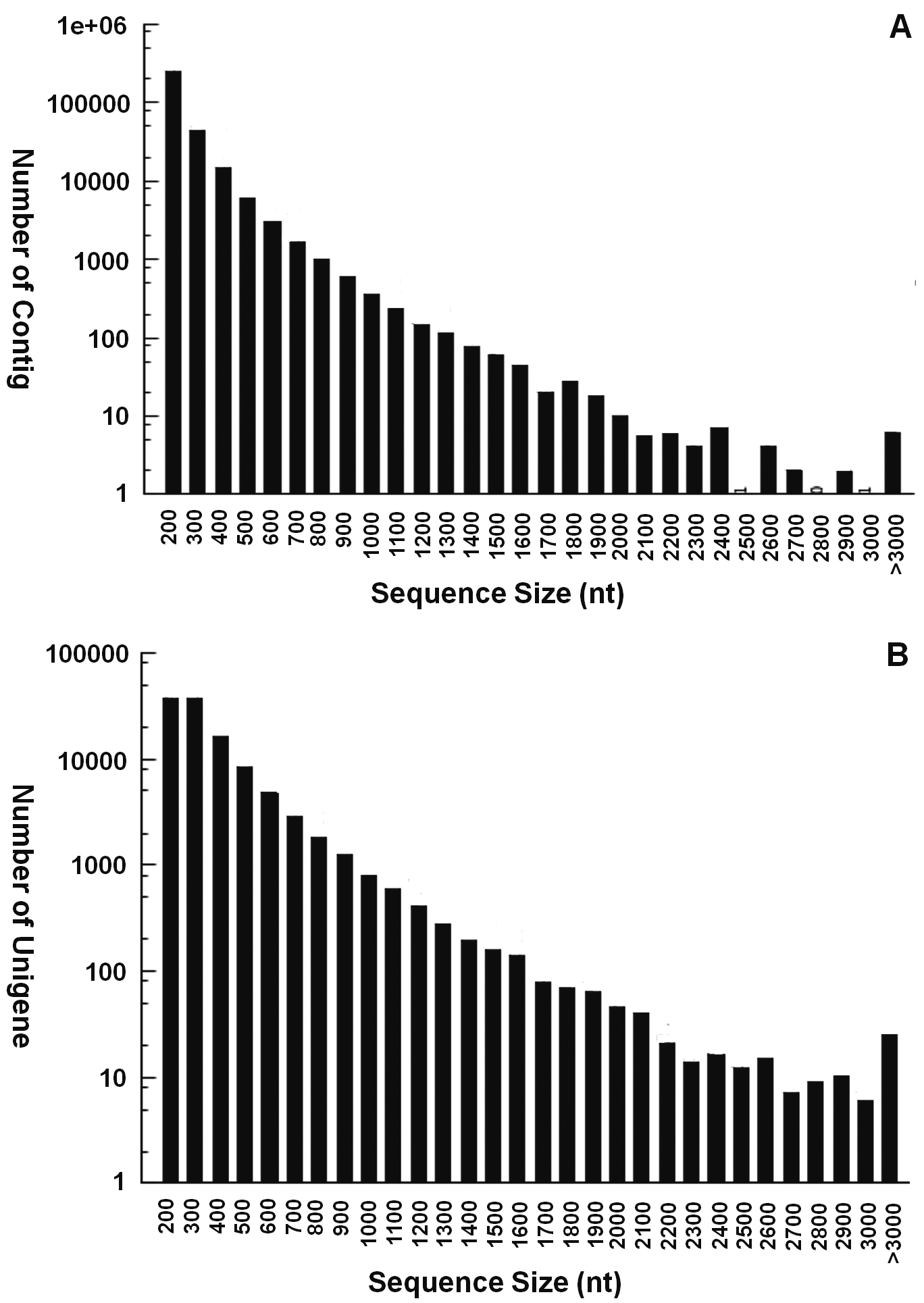

Supplement: Figure S1 — Summary of the C. amaranticolor transcriptomic sequences. (A) Size distribution of Illumina sequencing contigs. (B) Size distribution of distinct sequences after paired-end and gap filling. (TIF) [file pone.0045953.s001.tif]

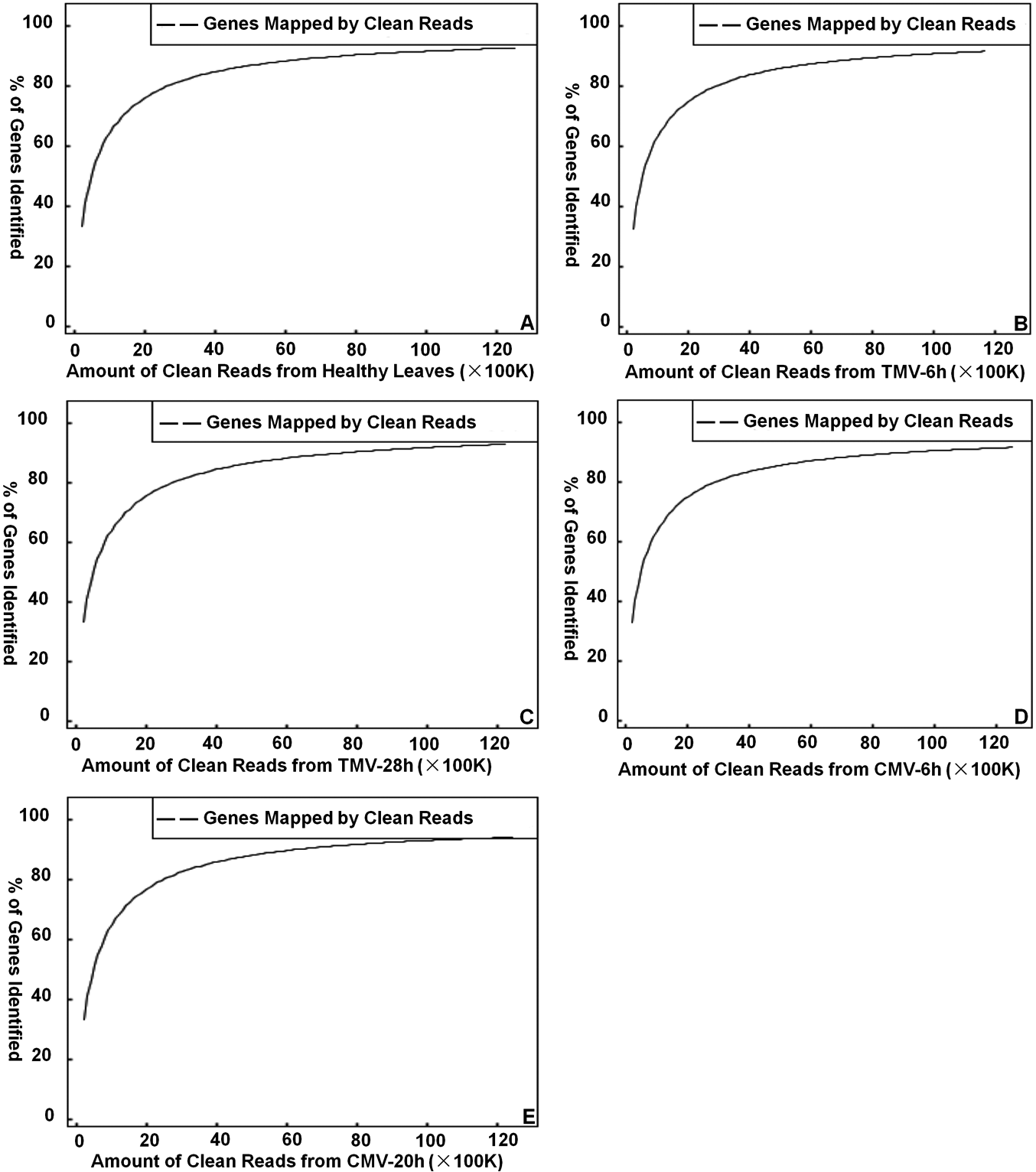

Supplement: Figure S2 — Relationship between the number of identified genes and the sequencing amount (total read number). The figures represent DGE reads of healthy leaves (A), TMV-6h (B), TMV-28h (C), CMV-6h (D) and CMV-20h (E), respectively, showing a trend of saturation. When the sequencing amount reaches 12 millions, the number of identified genes almost ceases to increase. (TIF) [file pone.0045953.s002.tif]

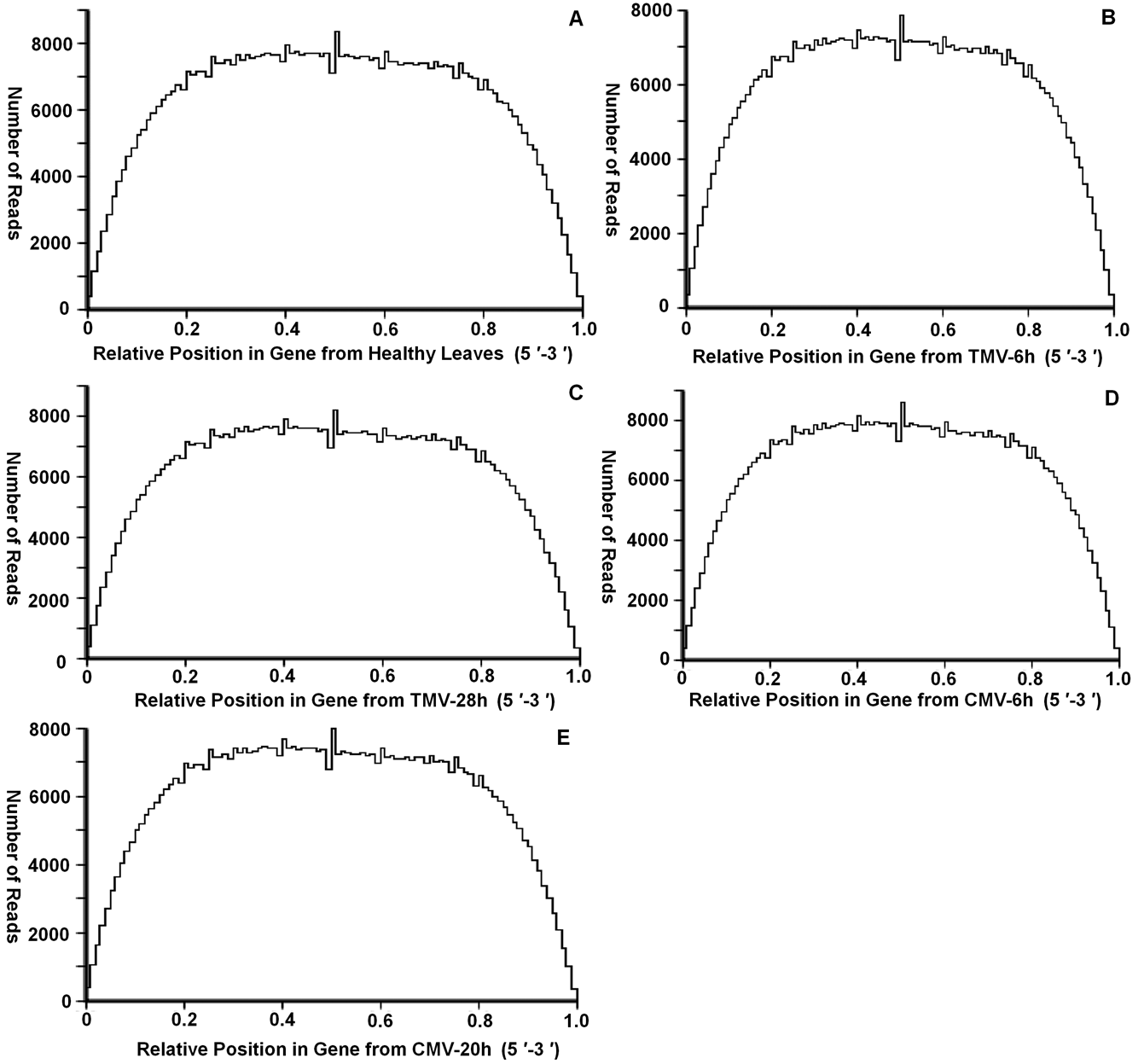

Supplement: Figure S3 — Distribution of DGE reads derived from healthy leaves (A), TMV-6h (B), TMV-28h (C), CMV-6h (D) and CMV-20h (E) on reference genes in the C. amaranticolor transcriptome. (TIF) [file pone.0045953.s003.tif]
